# Interplay of Porosity, Wettability, and Redox Activity as Determining Factors for Lithium–Organic Electrochemical Energy Storage Using Biomolecules

**DOI:** 10.1002/cssc.201903156

**Published:** 2020-03-05

**Authors:** Ivan K. Ilic, Milena Perovic, Clemens Liedel

**Affiliations:** ^1^ Department of Colloid Chemistry Max Planck Institute of Colloids and Interfaces Am Mühlenberg 1 14476 Potsdam Germany

**Keywords:** biomass, electrochemistry, energy conversion, polymers, redox chemistry

## Abstract

Although several recent publications describe cathodes for electrochemical energy storage materials made from regrown biomass in aqueous electrolytes, their transfer to lithium–organic batteries is challenging. To gain a deeper understanding, we investigate the influences on charge storage in model systems based on biomass‐derived, redox‐active compounds and comparable structures. Hybrid materials from these model polymers and porous carbon are compared to determine precisely the causes of exceptional capacity in lithium–organic systems. Besides redox activity, particularly, wettability influences capacity of the composites greatly. Furthermore, in addition to biomass‐derived molecules with catechol functionalities, which are described commonly as redox‐active species in lithium–bio‐organic systems, we further describe guaiacol groups as a promising alternative for the first time and compare the performance of the respective compounds.

## Graphical abstract

**Battery powered**: The influences of redox activity, the kind of functional group, and wettability are evaluated in biomass‐based materials for electrochemical energy storage.

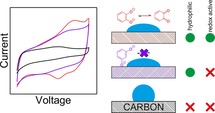

## Introduction

Guaiacol groups are common in biomolecules, both in small molecules such as vanillin as well as in macromolecules such as lignin.^1^ Although they are not redox‐active per se, irreversible oxidation in water results in the formation of the *o*‐quinone/*o*‐hydroquinone redox pair.^2^ This reaction was first applied prominently by Milczarek and Inganäs in cathode materials from lignin, which has abundant guaiacol units, for green electrochemical energy storage devices.^3^ Since then, many endeavors to improve lignin‐based cathodes have been undertaken.^4^, ^5^, ^6^, ^7^, ^8^, ^9^, ^10^, ^11^, ^12^ Additionally, cathodes based on polymers from biorefined monomers such as guaiacol and syringol,^13^ phenolic acids,^14^ and vanillin^15^ have been investigated in acidic aqueous electrolytes. Aqueous electrolytes are important for energy storage in these materials as the established process for the formation of quinones requires the presence of water.^2^ Recently, a lignin–PEDOT [PEDOT=poly(3,4‐ethylenedioxythiophene)] hybrid material was investigated as an electrode material in a lithium–organic system. However, lignin mainly serves to dope PEDOT in this system. PEDOT constitutes the majority of the electrode, which limits the sustainability of such a material.^16^

In polymer‐based electrodes, redox‐active polymers are always mixed with conductive additives such as carbon nanotubes,^17^ porous carbons,^9^, ^10^, ^15^, ^18^ and conductive polymers,^3^, ^19^ all of which contribute significant capacitive energy storage. Both cyclic voltammograms and galvanostatic measurements of the mixed cathode materials are used to show influences of both distinct redox‐active groups (high current only at distinct voltages in cyclic voltammetry and plateau‐like galvanostatic behavior) and capacitive behavior (rectangular cyclic voltammogram and triangular galvanostatic curve). Consequently, it is debatable whether the devices should be denoted as supercapacitors or batteries.^20^

In studies on organic electrode materials, conductive additives such as carbon materials are often considered passive parts of the electrode composition.^21^, ^22^ To describe charge storage in organic materials more thoroughly, sometimes electrodes without organic active materials are designed, and the resulting capacity is subsequently subtracted from the total capacity. However, such investigations suffer from the possible systematic error when neglecting the modification of charge storage properties of carbon additives by organic active materials.

The rapidly increasing number of publications on bio‐derived active electrode materials requires the discussion of such possible influences in more detail. Consequently, we investigate influences of different components in common biopolymer‐based materials for electrochemical energy storage. As model systems, we describe hybrid energy storage materials made from model biopolymers and carbon additives. In the former, several low‐molecular‐weight compounds, which have active functionalities common to biomolecules, are immobilized on a polymer backbone. For immobilization, we make use of the formation of Schiff bases, which can be used to graft aldehydes onto amine‐containing polymers, most notably, chitosan^23^ and polyallyamine.^24^, ^25^, ^26^, ^27^ Vanillin is investigated because it contains guaiacol groups. Protocatechuic aldehyde contains a catechol functionality, that is, it may be formed upon the demethylation of the guaiacol unit in vanillin. Finally, 3,5‐dihydroxzbenzaldehyde is investigated because it is an isomer of protocatechuic aldehyde. However, as the hydroxyl groups are found in the *meta* instead of the *ortho* position, is not redox‐active.

The processing of active polymeric materials with conductive additives, for example, by the preparation of a slurry that contains both components, spreading on a current collector, and drying, is a conventional way to create organic batteries. For the formation of electrodes, we chose microporous carbons as conductive additives because of the possibility to synthesize them from biowaste,^28^ which makes them an attractive sustainable conductive additive, and because of their intrinsically high capacitance that allows the formation of hybrid electrochemical energy storage devices.^29^ The contributions of carbon, the wettability of the electrolyte, and different functional units to the resulting lithium‐ion‐based energy storage devices are discussed. Firstly, we characterize the polymers under investigation and then their composites with microporous carbon to assess the influence of different physical properties and functionalities on charge storage. Finally, the electrochemical performance of these composites are investigated and compared.

## Results and Discussion

### Synthesis of polymers and their hybrid materials

**Scheme 1:**
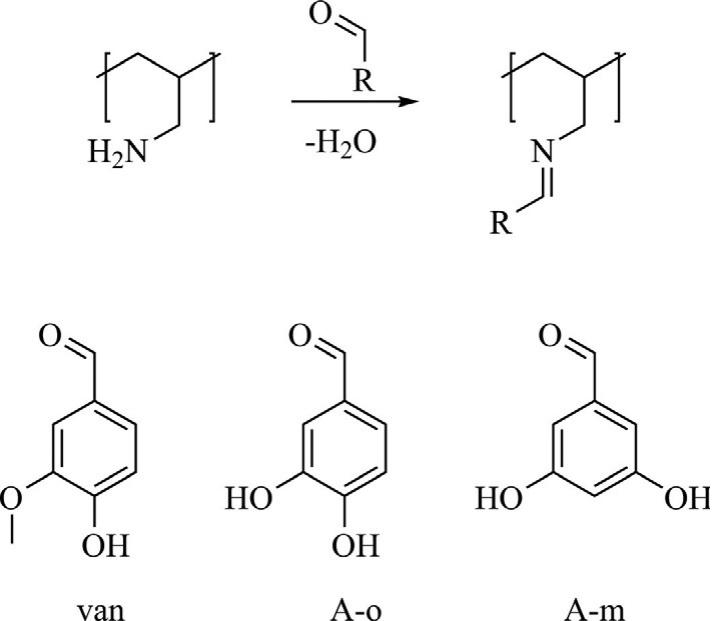
Reaction of polyallylamine with different aldehydes.

Schiff bases of polyallylamine (PAAm) and different aldehydes, namely, vanillin (van), protocatechuic aldehyde (which features two phenolic groups in the *ortho* position; A‐o), and 3,5‐diyhdroxybenzaldehyde (which features two phenolic groups in the *meta* position; A‐m), were synthesized in ethanolic solutions as described in the Experimental Section and Supporting Information (Scheme 1). The addition of aldehydes to PAAm results in an immediate color change as expected for the formation of Schiff bases, followed by precipitation (Figure S1) caused by the interaction of leftover amine groups with acidic phenolic groups.^24^ During subsequent drying, the formation of Schiff bases proceeds,^30^ and washing with anhydrous ethanol yields the pure modified polymers (denoted P‐van, P‐o, and P‐m, respectively).

**Figure 1:**
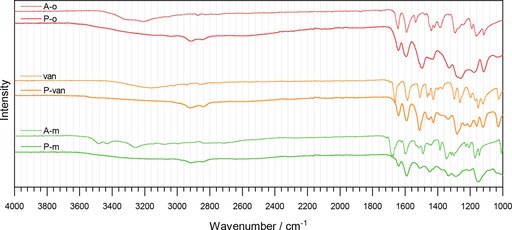
IR spectra of A‐o, van, A‐m, and their corresponding Schiff bases with PAAm in the range of 1000–4000 cm^−1^.

The successful formation of Schiff bases is confirmed by using IR spectroscopy (Figure 1), as the resulting polymers contain the functional groups from the respective phenyl ring. Broad bands above $\tilde{\nu }$ =3000 cm^−1^, from hydroxyl groups, are retained both in the spectra of the polymers and aldehydes, which confirms the retention of the hydroxyl groups during synthesis. Two new bands appear in the spectra of polymers at approximately $\tilde{\nu }$ =2840 and 2920 cm^−1^, which indicates C−H stretching vibrations from the polyallylamine backbone.^31^ No aldehyde groups are detected in the spectra of the polymers, but new bands at approximately $\tilde{\nu }$ =1680 cm^−1^ represent imine functionalities in all three samples, which confirms both the successful reaction and the successful removal of any unreacted aldehydes.^32^ Intriguingly, the shift between the imine and aldehyde peak is very pronounced in the case of van/P‐van and A‐m/P‐m but is rather subtle in the case of A‐o/P‐o, only by a few cm^−1^, which originates from the different wavenumber of the aldehyde carbonyl vibrations in A‐o, van, and A‐m. The substitution rates in the samples (details are explained in the Supporting Information) calculated using the amount of N determined by using elemental analysis is summarized in Table 1. Substitution rates above 80 % confirm the successful grafting of aldehydes onto PAAm to yield polymers with a high density of redox‐active or comparable functional groups. Notably, the apparent substitution rate of P‐m, which exceeds 100 %, is a result of the limitations of this method of analysis, but other methods unfortunately also overestimated the substitution rates of Schiff bases on PAAm in the past.^24^, ^26^ This overestimation could be ascribed to a small amount of water present in the samples, which is visible from the results obtained from the polymers by using thermogravimetric analysis (TGA; Figure S2). Notably, several indications such as the deviation between multiple measurements and the occurrence of sulfur in the samples show that these results are not precise. The use of these methods is to ensure comparability between the samples, which they do. Similar substitution rates allow us to compare differences caused by the redox‐active and ‐inactive functional groups.

**Table 1:** Substitution rates of PAAm–aldehyde Schiff bases.

Polymeric Schiff base	Amt. of N [wt %]^[a]^	Substitution rate [%]^[a]^
P‐o	8.69–8.98	82.4–86.7
P‐van	7.78–7.80	91.3–91.7
P‐m	7.70–7.72	103.6–103.9

[a] Details on the calculation are given in the Supporting Information. The values represent data from two separate measurements.

We formed hybrid cathode materials by combining the polymers and carbon as explained in the Supporting Information and label them C/x in which x is the corresponding polymer. SEM was used to show the morphology of the obtained materials before [C(pristine)] and after ball‐milling (C and C/P‐o; Figure S3). Upon prolonged milling, the big particles of conductive carbon become smaller. The morphologies of both C/P‐o and the neat carbon after ball‐milling are similar because of the tight composite between the polymer and the conductive carbon. We used energy‐dispersive X‐ray mapping to show a homogeneous dispersion of C, O, and N (Figure S4).

### Comparison of the electrochemical performance of redox‐active and ‐inactive polymers

**Figure 2:**
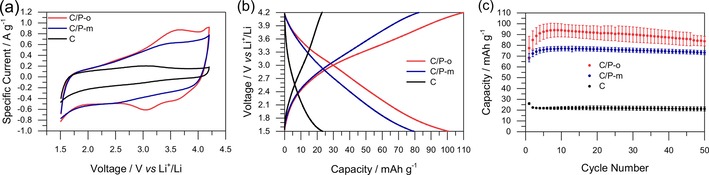
Comparison of the electrochemical performance of C/P‐o, C/P‐m, and porous carbon (C). The tests were performed in a lithium half‐cell setup with lithium as counter electrode and 1 m LiPF_6_ in EC/DEC (1:1) as electrolyte. a) CVs at 5 mV s^−1^, 10^th^ cycle. b) Charge–discharge tests at 0.2 A g^−1^, 10^th^ cycle. c) Discharge capacities calculated from the charge–discharge tests at 0.2 A g^−1^.

C/P‐o, C/P‐m, and microporous carbon are compared in Figure 2 in terms of their electrochemical performance during cyclic voltammetry (CV) and charge–discharge tests. The redox activity of C/P‐o (Scheme S2) is clearly detectable in the CV from peaks at approximately 3.3 V versus Li^+^/Li (Figure 2 a). In the low‐voltage region, the voltammograms of C/P‐o and C/P‐m match fairly well, but there are no redox peaks at approximately 3.3 V versus Li^+^/Li observable in the trace of C/P‐m, as expected for redox‐inactive species. Similarly, the absence of redox peaks is characteristic of purely microporous carbon.

The capacity of microporous carbon in galvanostatic charge–discharge experiments is very low. Upon the addition of P‐m and even more so upon the addition of P‐o (C/P‐m and C/P‐o, respectively), the capacity increases significantly. Upon prolonged cycling, C and C/P‐m retain their capacity, whereas C/P‐o shows subsequent decay (Figure 2 c), which will be discussed later. The higher capacity of C/P‐o than C/P‐m can be explained easily by the redox activity of P‐o that results in a slightly bell‐shaped galvanostatic discharge behavior of C/P‐o (Figure 2 b) and a significantly higher capacity. The range of the increased discharge capacity in C/P‐o compared to that of C/P‐m (nonparallel discharge curves shown in Figure 2 b) at approximately 2.7–3.8 V versus Li^+^/Li matches the location of the discharge peaks in the CVs of C/P‐o (Figure 2 a). Peaks in the latter correspond to the redox reaction between catechol and *o*‐hydroquinone groups, which is assigned to increased capacity (C/P‐o compared to C/P‐m) in Figure 2 b because of the influence of redox reactions. No belly‐shaped behavior can be observed for C and C/P‐m (Figure 2 b). A comparison of the electrochemical impedance spectra (EIS) of C/P‐o, C/P‐m, and C (Figure S6) shows the similar behavior of the composites. The Nyquist plots show the good conductivity and permeability of all the samples as expected, and C has the fastest kinetics as no polymer blocks its pores.

**Figure 3:**
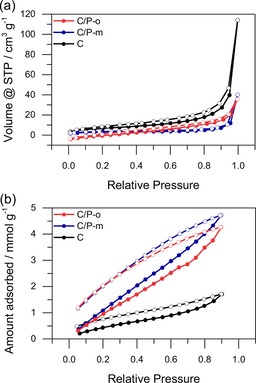
a) N_2_ (77 K) and b) water vapor (298 K) physisorption isotherms of C/P‐o, C/P‐m, and C. Full dots represent adsorption, and empty ones represent desorption.

However, the significantly higher capacity observed if we compare C/P‐m and microporous carbon cannot be explained by redox reactions as P‐m is not redox‐active. A possible explanation for this may be the increased wettability of the carbon surfaces by the electrolyte after functionalization with the heteroatom‐rich polymer. Consequently, next we discuss the N_2_ and water vapor physisorption behavior to assess the hydrophilicity of the prepared hybrid materials and, therefore, their wettability with solvents of high polarity, such as electrolytes (Figure 3).

During ball‐milling, C loses most of its internal surface area, probably caused by a combination of high‐energy ball‐milling and the blocking of the pores by the binder (cf., physisorption behavior of the pristine microporous carbon in Figure S7). The Brunauer–Emmett–Teller (BET) surface area of the hybrid materials with the model polymers (C/P‐o and C/P‐m) are even lower than that of C, possibly because of the relatively low amount of microporous carbon in the hybrid material and because of the additional blocking of the pores by the active polymers. All samples exhibit high external surface areas because of the loose packing of particles.

Water vapor physisorption does not follow the same trend as N_2_ physisorption. The water vapor uptake in C/P‐o and C/P‐m is similarly high, and the carbon material adsorbs significantly less water vapor than C/P‐o and C/P‐m over the entire range of relative pressure. As the elemental composition of P‐o and P‐m is the same, this behavior may be ascribed to abundant hydroxyl groups in the polymers, which results in mixed carbon‐polymer materials with a similar hydrophilicity. Hydrophilicity is significantly higher in the hybrid materials than in the carbon material, and subsequently, as a result of the high polarity of water and electrolytes, so is the wettability by polar electrolytes. As the electrolyte reaches a larger part of the internal surface of the microporous carbon, the contribution to energy storage by the formation of the electric double layer increases. More charge can be stored on the surface for both C/P‐o and C/P‐m compared to carbon, which leads to a higher capacity. Similar behavior was described previously in aqueous solutions of polypyrrole with and without chitosan, in which the redox‐inactive chitosan increased the capacity of polypyrrole significantly.^33^ Notably, ball‐milling tends to increase the hydrophilicity of carbon materials, although to a low extent.^34^

### Comparison of the electrochemical performance of catechol and guaiacol groups

**Figure 4:**
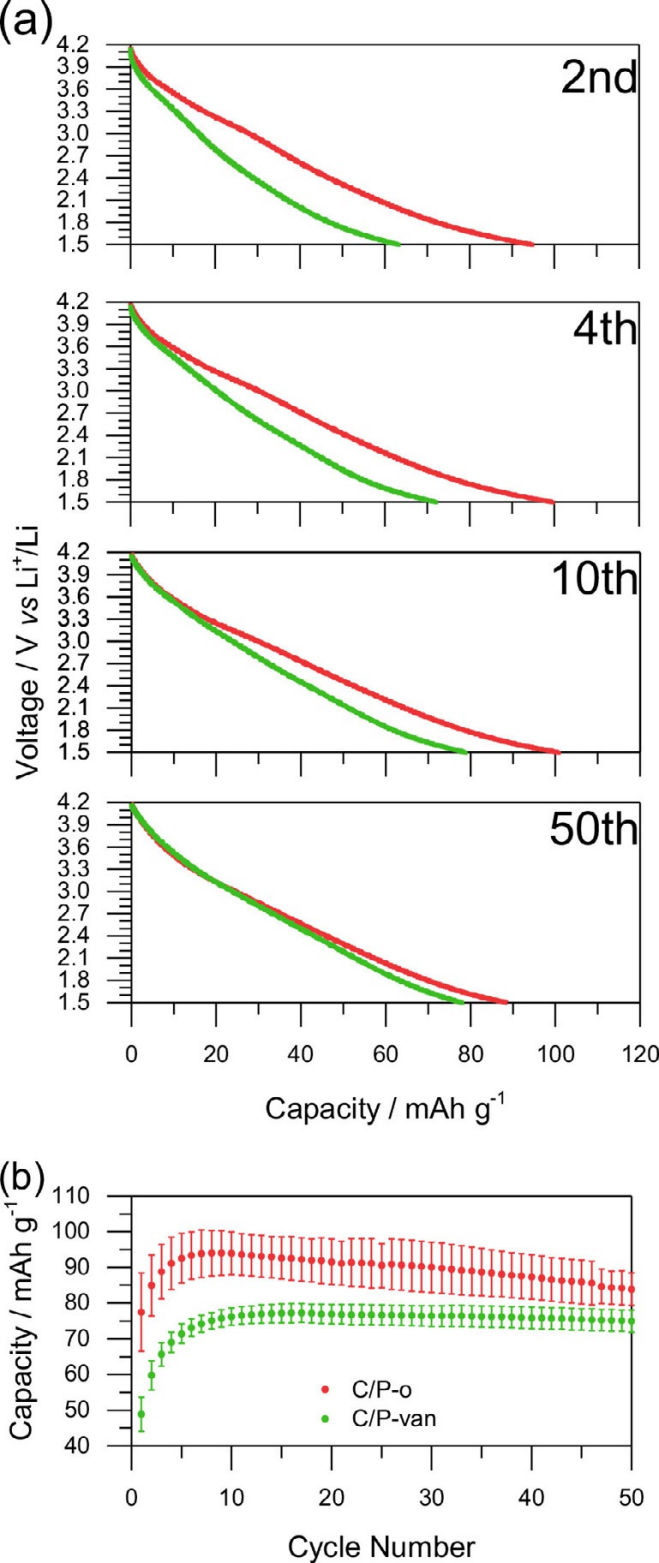
Charge–discharge tests of C/P‐o and C/P‐van. The tests were performed in a lithium half‐cell setup with lithium as the counter electrode and 1 m LiPF_6_ in EC/DEC (1:1) as the electrolyte. a) Discharge curves at 0.2 A g^−1^ at different cycles, for which charge and discharge were performed between 4.2 and 1.5 V. b) Discharge capacities at 0.2 A g^−1^.

Catechol groups in hybrid materials with microporous carbons contribute to the overall capacity both through the increased wettability of carbon surfaces by the electrolytes and redox activity. Still, catechol groups are rather rare in nature, notable exceptions are dopamine and tannic acid, which have been described previously for electrochemical energy storage.^35^, ^36^, ^37^, ^38^, ^39^, ^40^ In contrast, guaiacol groups (which are chemically similar; cf. Scheme 1) are abundant in nature, for example, in low‐value biogenic materials such as lignin. To our knowledge, such materials have never been used in secondary lithium ion batteries without additional redox active polymers to date. Thus, we will focus on such materials by comparing the electrochemical performance of C/P‐o and C/P‐van (Figure 4). Initially, the discharge curves differ greatly, both in terms of overall capacity as well as shape, but become more similar with the increase of the number of cycles. Unlike that of C/P‐o, the discharge curve of C/P‐van shows no belly shape at the beginning. With the increase of the number of cycles, the shape changes, and ultimately, almost matches that of the C/P‐o discharge curve after approximately 50 charge–discharge cycles. The same behavior is apparent in the CV curves (Figure S8). Although no clear redox peaks are observed in the early cycles of C/P‐van, such peaks at similar potential as seen in the CV of C/P‐o appear with continuing cycling, and the CVs of C/P‐van and C/P‐o start to resemble each other. We performed EIS for both samples (Figure S6), which shows the good conductivity of the materials, and C/P‐van exhibits slightly slower kinetics possibly because of the presence of methoxy groups.

In aqueous systems,^2^ demethylation occurs in the first oxidation step, expressed by the high current in the CV curves at voltages approximately 0.2 V higher than the reversible redox peak that evolves in the next reduction cycle. A quinone–hydroquinone redox pair thus can be formed easily also in molecules that initially contain guaiacol groups. As this mechanism requires the presence of water, it is impossible in a lithium‐ion‐containing setup in water‐free organic electrolytes. Consistently, no such first irreversible oxidation peak is observable by using CV in our setup. Still, galvanostatic and CV curves of C/P‐van indicate the formation of the same quinone–hydroquinone redox couple as that in C/P‐o with prolonged cycling (Figure 4 and Figure S8) probably because of the slow demethylation that exposes redox‐active functionalities. The mechanism for the involved demethylation process is unclear; however, we suggest a similar mechanism to that found in aqueous systems, with the difference that fluoride anions serve as nucleophiles instead of water and form fluoromethane in the process. As no significant irreversible oxidation peak is observed in a distinct early CV cycle (Figure S9 a and b), unlike in the case of similar polymers in aqueous systems (Figure S9 c), we conclude that demethylation requires several charge–discharge cycles in a lithium half‐cell setup. Irreversible oxidation, which usually results in diminishing capacity with prolonged cycling, is common in organic battery materials.^18^ Notably, C/P‐van reaches constant capacity after approximately 10 charge–discharge cycles. After the maximum was reached, the capacity was constant for 50 charge–discharge cycles, in contrast to the case of C/P‐o for which the capacity decreases slowly immediately after it reached the maximum. The behavior of both during continuous charge and discharge can be explained by a model in which both materials pass through three phases: sleeping, living, and dead phases as established for lithium–sulfur batteries by Risse et al.^41^ In our case, the sleeping phase represents all the guaiacol groups that did not yet undergo initial oxidation and all the hydroquinone groups that are unreachable to the electrolyte. The living phase represents all the quinone–hydroquinone redox pairs that undergo redox reactions in that step and is the only phase that contributes to the Faradaic charge storage of the material. Finally, the dead phase represents all the degraded groups that cannot undergo a redox reaction anymore. Furthermore, slight solubility in the electrolyte and the decomposition of the polymer by traces of water may contribute to the slow capacity fading in the dead phase. During cycling, units can change irreversibly from the sleeping to living phase or from the sleeping/living to dead phase.

The different charge storage behavior with continuous cycling may be explained by differences in the sleeping phases between both materials. The oxidation of hydroquinone groups happens much faster than that of guaiacol groups, which results in the complete transition of C/P‐o from the sleeping phase to the living phase within a few cycles. The subsequent fading capacity indicates a slow transition to the dead phase. In contrast, the constant capacity of C/P‐van after the maximum is reached, which may be ascribed to the continuous slow transition of some guaiacol groups from the sleeping to living phase at a similar rate as the transition of other active groups from the living phase to the dead phase. Consequently, this does not necessarily indicate the higher stability of the material during cycling. Ultimately, the maximum capacity is lower in C/P‐van than C/P‐o as partial demethylation (formation of the living species) continues after the maximum capacity is reached. Only after approximately 50 cycles do the capacities of C/P‐m and C/P‐o become similar because of the formation of quinone groups in C/P‐van and the irreversible oxidation of C/P‐o. The final apparent specific capacity of C/P‐van is still slightly lower than that of C/P‐o because the specific capacity is calculated from the initial mass of the hybrid material, which includes the methyl groups in P‐van. The demethylation of guaiacol groups reduces the weight of the polymer material slightly, which makes the assumed gravimetric capacity an underestimation.

### New promising sustainable cathode material: C/P‐o

We next evaluate the electrochemical behavior of the best‐performing material C/P‐o in detail. If we compare the charge storage behavior of C/P‐o, C/P‐m, and C (Figure 2 b), the influences of carbon, redox activity, and changes in wettability may be estimated as discussed in more detail in the Supporting Information. According to the galvanostatic charge–discharge experiments, after 10 cycles, reversible redox reactions contribute to 18.1 % of the charge storage of the total electrode material. With only 13.0 % derived from capacitive charge storage on the surface of the unmodified carbon material, changes in wettability of the surface have a major influence on charge storage (68.9 %). Abundant phenolic functionalities and imines result in significantly enhanced hydrophilicity, which facilitates capacitive charge storage using polar electrolytes. These results point to the importance of the selection of appropriate binders and carbons in the design of electrode materials from renewable resources.

**Figure 5:**
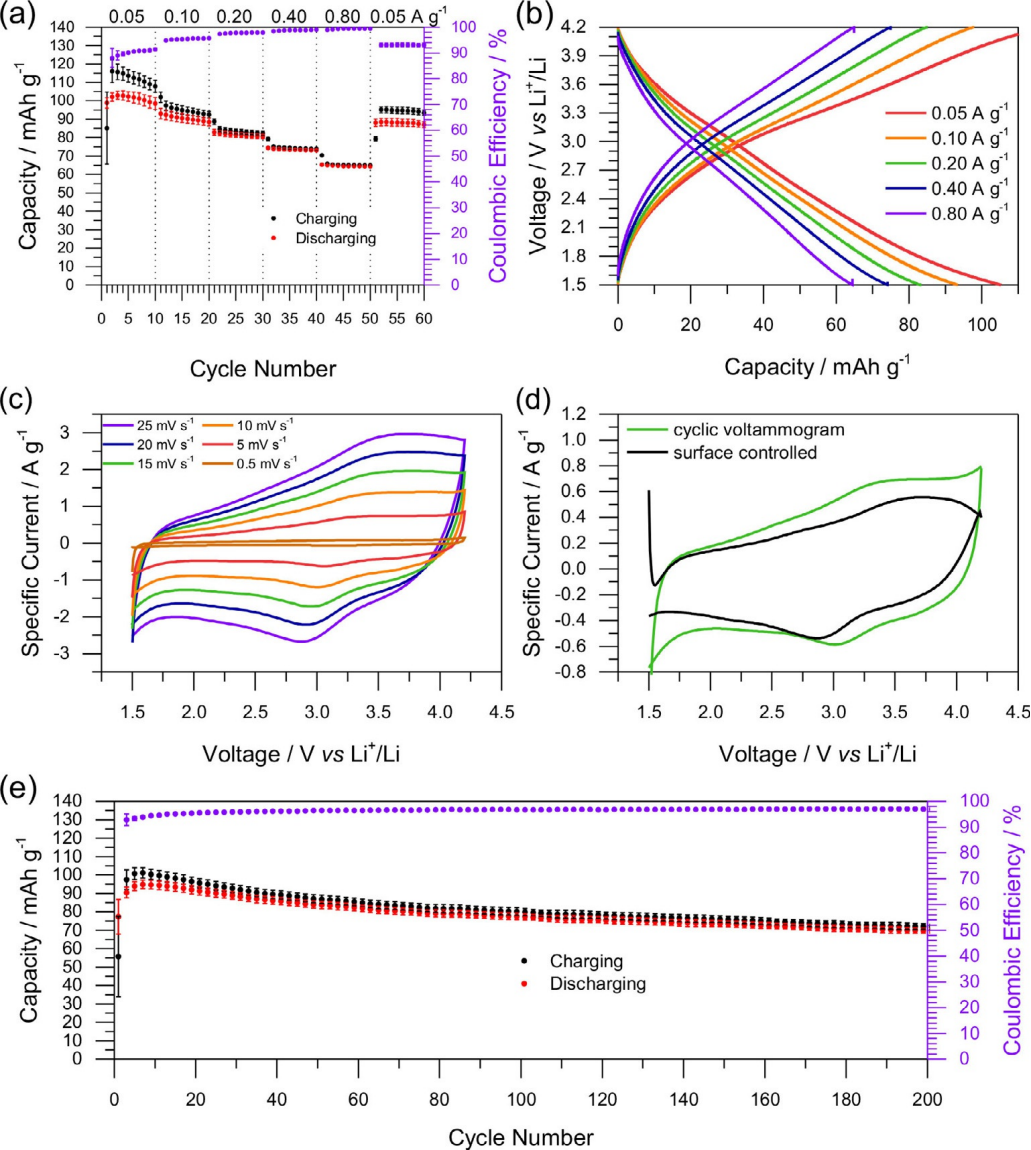
Detailed analysis of the electrochemical performance of C/P‐o. The test was performed in a lithium half‐cell setup with lithium as the counter electrode and 1 m LiPF_6_ in EC/DEC (1:1) as the electrolyte. a) Charge–discharge tests at different current densities as indicated. b) Galvanostatic charge–discharging curves (5^th^, 15^th^, 25^th^, 35^th^, and 45^th^ cycle from a). c) CV at different rates as indicated. d) Diffusion‐controlled processes at 5 mV s^−1^ as calculated from the CV at different rates (see Supporting Information for details). e) Long‐term stability test at 0.1 A g^−1^ (every third data point is shown).

Further information on the electrochemical behavior of C/P‐o is given in Figure 5. We performed charge–discharge tests at different current densities (Figure 5 a) between 0.05 and 0.80 A g^−1^ (see Experimental Section for details). Within the first three cycles, the capacity increases and starts to decrease slowly afterwards, as discussed above. At higher charge–discharge rates up to 0.80 A g^−1^, the capacity decreases only moderately and is restored to almost initial values after the current density returns to 0.05 A g^−1^, which prominently supports diffusion‐ and surface‐controlled charge storage. Discharge curves at different current densities (Figure 5 b) generally appear as almost straight lines with a slight indication of a belly shape, which indicates dominant capacitive behavior with some added distinct quinone–hydroquinone redox reactions, respectively.

CV curves of C/P‐o at different rates are presented in Figure 5 c. As the integrated area of the CV curve increases slowly over some tens of cycles (Figure S10), for better comparability the hybrid material was cycled firstly for 100 cycles at 25 mV s^−1^ followed by cycling at 20, 15, 10, 5, and 0.5 mV s^−1^ for one cycle each. CV curves show clear oxidation and reduction peaks at 3.6 and 2.8 V, respectively, which match the range of the belly‐shaped behavior in the galvanostatic charge–discharge curves. From the performance at different cycling speeds, the ratio of Faradaic charge storage and double layer capacitance was calculated (details can be found in the Supporting Information).^42^, ^43^, ^44^, ^45^ Except for the limits between which the CV experiments were performed, the resulting curve for the capacitive contribution to charge storage (Figure 5 d, black curve) resembles the CV of C/P‐m (Figure 2 a, blue curve). In both cases, the capacity is approximately 80 % as high as that of C/P‐o (84.8 % cf. Figure 2 a, red curve and 74.8 % cf. Figure 5 d green curve, respectively), which confirms the importance of hydrophilicity on the charge storage and matching the results of galvanostatic experiments (redox reactions in C/P‐o are responsible for 18.1 % of the charge storage). Clearly, the addition of P‐o to carbon not only introduces the possibility of Faradaic charge storage but also facilitates capacitive charge storage. The notable differences between the calculated capacitive contribution to charge storage shown in Figure 5 d and the charge storage in C/P‐m (Figure 2 a, blue curve) at low and high voltages can be ascribed to the limitations of the assumed model, expressed by a low coefficient of determination (*R* ^2^; Figure S11) caused by the sudden increase or decrease in current density at the edges of the CVs. Slight differences between the capacitive charge storage in C/P‐m and calculated capacitive contribution to C/P‐o as denoted above can be ascribed primarily to the aforementioned collapse of the model at the edges of the CVs.

During long‐term cycling at 0.1 A g^−1^ (Figure 5 e), the capacity decreases slowly as is observed generally for organic systems (see discussion above). However, a remarkable retention of 73.5 % is observed after 200 cycles (on average 0.13 % decay of capacity per cycle). The high Coulombic efficiency of approximately 97 % makes P‐o a promising polymer for electrochemical energy storage applications, comparable to other biomass‐derived batteries.^46^, ^47^

## Conclusions

Polymers made from polyallylamine and redox‐active and ‐inactive aldehydes were used as model compounds for biogenic polymers to elucidate the different contributions to electrochemical energy storage in future bio‐based batteries and lithium ion capacitors. In the systems under investigation, which included carbon, binder, and the (in)active polymer, only 18.1 % of the capacity could be ascribed to distinct redox reactions. All samples showed a significantly higher capacity than microporous carbon itself, and the difference was ascribed to the increased hydrophilicity of the capacitive hybrid material that causes better wettability by the electrolyte and better lithium transport behavior. Therefore, we conclude that a significant portion of the capacity of electrochemical energy storage devices based on natural polyphenols stems from capacitive charge storage on hydrophilic surfaces in addition to redox activity. Furthermore, the electrochemical performance in a lithium‐ion‐containing setting for the model polymers that contain redox‐active catechol groups was compared to that with naturally abundant guaiacol groups. Both showed a similar performance after prolonged cycling, with a slower initial increase in capacity for the guaiacol‐containing polymers because of their slow demethylation processes. From these findings, a biomass‐derived hybrid material was prepared that showed excellent properties for electrochemical energy storage in lithium–organic systems with a capacity of over 100 mA h g^−1^ at 0.05 A g^−1^ and capacity retention of 73.5 % after 200 cycles at 0.1 A g^−1^.

## Experimental Section

### Materials

Protocatechuic aldehyde (A‐o, Carl Roth), vanillin (van, Sigma–Aldrich), 3,5‐dihydroxybenzaldehyde (A‐m, Fischer), an aqueous solution (15 %) of polyallylamine with an average molecular weight of 15 000 g mol^−1^ (PAAm, Polysciences, Inc.), carbon EQ‐AB‐520Y (C, MTI Corporation), carbon paper Spectracarb 2050A‐0550 (Fuel Cell Store), and the solvents ethanol (Fischer) and *N*‐methyl‐2‐pyrrolidone (NMP, PanReac AppliChem) were used as received.

### Synthesis of P‐o^24^

An aqueous solution of PAAm (0.33 g, 0.87 mmol of repeating units) was added to ethanol (10 mL) and stirred for 10 min. After the addition of A‐o (0.12 g, 0.87 mmol) dissolved in ethanol (10 mL), the mixture was stirred for 1 h. Afterwards the solvent was removed at 60 °C at a pressure of 180 mbar, which was lowered to 150 mbar shortly after the beginning of the solvent removal. After the obtained polymer was dried at 80 °C under vacuum for 2 h, it was washed with ethanol by centrifugation (4 times with 50 mL ethanol, 4000 rpm, for 5 min). The sample was dried in a vacuum oven overnight. More information on the synthesis in included in the Supporting Information.

### TGA and combustive elemental analysis

TGA measurements were performed by using a NETZSCH TG 209F1 Libra machine under N_2_ at a heating rate of 10 K min^−1^. Combustive elemental analysis was performed by using a varioMicro CHNS machine.

### Morphology characterization

The morphology at the surface was investigated by SEM by using a Zeiss Leo Gemini 1550 microscope, and the atomic distribution of C, O, and N was mapped by using energy‐dispersive X‐ray spectroscopy (EDX, X‐Max, Oxford instruments).

### Physisorption

Before the physisorption measurements, the samples were degassed under vacuum at 80 °C for 16 h. N_2_ physisorption measurements were performed by using a Quantachrome Quadrasorb SI physisorption instrument at 77 K, whereas water vapor physisorption was measured by using a Quantachrome Autosorb IQ physisorption instrument at 298 K.

### Spectroscopy

FTIR spectroscopy was performed by using a Nicolet iS 5 FTIR spectrometer (ThermoFisher Scientific).

### Electrochemical measurements

All electrochemical measurements were performed in Swagelok‐type cells and tested using a BioLogic MPG2 potentiostat. Lithium foil, Celgard 2325 (13 mm in diameter, 25 μm thick), and 1 m LiPF_6_ in ethylene carbonate (EC) and diethyl carbonate (DEC; volumetric ratio 1:1, 100 μL; solution used as obtained from Sigma–Aldrich) were used as counter electrode, membrane, and electrolyte, respectively. A circular carbon current collector covered with the active material was used as the working electrode. The electrolyte was chosen as it is one of the most common choices for carbonyl‐based organic lithium‐ion batteries.^48^ All Swagelok‐type cells were assembled in a glovebox with low water and O_2_ levels. Charge–discharge measurements were performed at a constant current density (0.2 A g^−1^ for 50 cycles and 0.1 A g^−1^ for 200 cycles) or at varying current density (20 cycles at 0.05 A g^−1^, 10 cycles at 0.10 A g^−1^, 10 cycles at 0.20 A g^−1^, 10 cycles at 0.40 A g^−1^, 10 cycles at 0.80 A g^−1^) in triplicate. CV was performed by cycling at 25 mV s^−1^ for 100 cycles and at 20, 15, 10, 5, and 0.5 mV s^−1^ subsequently for one cycle each. In the displayed CV curves, the last cycle at the respective speed is shown unless mentioned otherwise.

## Conflict of interest

*The authors declare no conflict of interest*.

## Supporting information

As a service to our authors and readers, this journal provides supporting information supplied by the authors. Such materials are peer reviewed and may be re‐organized for online delivery, but are not copy‐edited or typeset. Technical support issues arising from supporting information (other than missing files) should be addressed to the authors.

Supplementary
