# Supplementary material for: Interplay of Porosity, Wettability, and Redox Activity as Determining Factors for Lithium–Organic Electrochemical Energy Storage Using Biomolecules
Source: ChemSusChem. 2020 Mar 5;13(7):1856–63. doi: 10.1002/cssc.201903156 (PMC7186837; doi:10.1002/cssc.201903156)
Supplement: Supplementary file 1 — Supplementary [file CSSC-13-1856-s001.pdf]

CHEMISTRY & SUSTAINABILITY
CHEMSUSCHEM
ENERGY & MATERIALS

Supporting Information

Interplay of Porosity, Wettability, and Redox Activity as Determining Factors for Lithium–Organic Electrochemical Energy Storage Using Biomolecules

Ivan K. Ilic, Milena Perovic, and Clemens Liedel*[a]

cssc_201903156_sm_miscellaneous_information.pdf

Synthesis of P-van[1]

An aqueous solution of PAAm (0.33 g, 0.87 mmol of repeating units) was added to ethanol (10 mL) and stirred for 10 minutes. After the addition of A-van (0.13 g, 0.87 mmol) dissolved in ethanol (10 mL) the mixture was stirred for 1 h. Afterwards the solvent was removed at 60 °C at a pressure of 180 mbar which was lowered to 150 mbar shortly after the beginning of the solvent removal. After drying of the obtained polymer at 80 °C under vacuum for 2 h, it was washed with ethanol using centrifugation (4 times with 50 mL ethanol, 5 min). The sample was dried in vacuum oven overnight.

Synthesis of P-m[1]

An aqueous solution of PAAm (0.33 g, 0.87 mmol of repeating units) was added to ethanol (10 mL) and stirred for 10 minutes. After the addition of A-m (0.16 g, 0.87 mmol) dissolved in ethanol (10 mL) the mixture was stirred for 1 h. Afterwards the solvent was removed at 60 °C at a pressure of 180 mbar which was lowered to 150 mbar shortly after the beginning of the solvent removal. After drying of the obtained polymer at 80 °C under vacuum for 2 h, it was washed with ethanol using centrifugation (4 times with 50 mL ethanol, 5 min). The sample was dried in vacuum oven overnight.

Synthesis of hybrid materials of P-x with carbon black

P-x (24 mg) and conductive carbon (30 mg) were ball milled for 50 min in a stainless steel jar before a solution of PVDF in NMP (6 mg mL^{-1}) was added (1 mL) and the mixture was ball milled for additional 10 min. The slurry (30 µL) was spread across a circular piece of carbon paper (11 mm in diameter) and dried at 80 °C for 17 h. For physisorption measurements, the slurry was spread in a petri dish before drying. The collected powder was later washed twice with ethanol (50 mL each time) and dried for 3 additional hours.

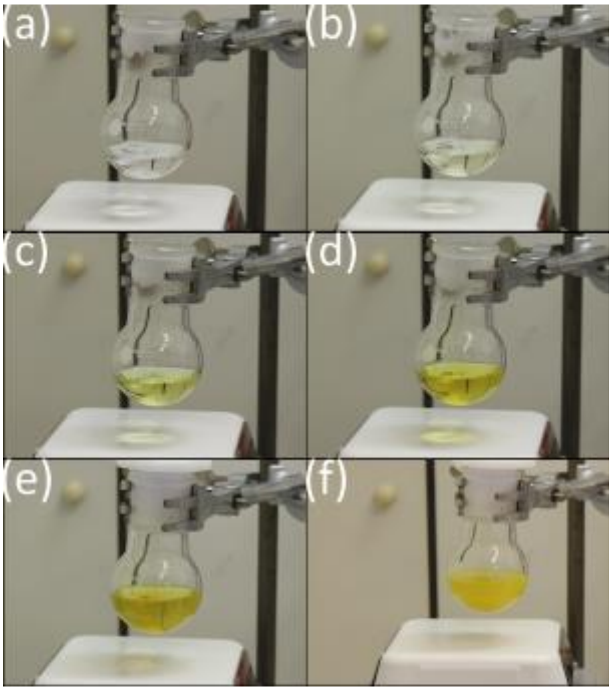

Figure S1. Reaction of P-o. Before adding the A-o containing solution (a), after adding 10% (b), 20% (c), 50% (d) and 100% (e) of the A-o containing solution, and after additional stirring for 1 h (f).

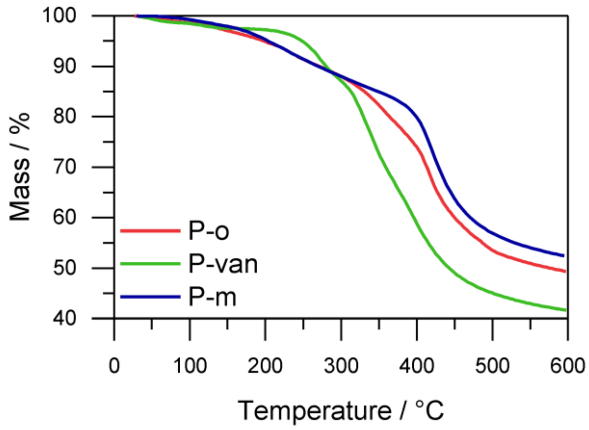

Figure S2. TGA of P-o, P-van and P-m.

Table S1. Elemental analysis of P-x.

	N [wt %]	C [wt %]	H [wt %]	S [wt %]
**P-o**	8.98	64.87	7.061	1.023
	8.69	65.21	6.944	0.914
**AVERAGE**	8.84	65.04	7.003	0.969
**P-van**	7.78	67.32	7.180	0.815
	7.80	67.18	7.186	0.773
**AVERAGE**	7.79	67.25	7.183	0.794
**P-m**	7.70	65.66	6.476	0.907
	7.72	65.69	6.620	0.360
**AVERAGE**	7.71	65.68	6.548	0.634

The substitution rate was determined from nitrogen content according to the equation:

$$w_N = \frac{m_N}{m_{polymer}} = \frac{Mr_N}{Mr_{PAAm} * (1 - \mu) + Mr_{P-x} * \mu}$$

$$\frac{Mr_N}{w_N} = Mr_{PAAm} - Mr_{PAAm} * \mu + Mr_{P-x} * \mu$$

$$\frac{Mr_N}{w_N} - Mr_{PAAm} = \mu * (Mr_{P-x} - Mr_{PAAm}) = \mu * (Mr_{A-x} - Mr_{water})$$

$$\mu = \frac{\frac{Mr_N}{w_N} - Mr_{PAAm}}{Mr_A - Mr_{water}}$$

where μ is the substitution rate, w_N is the nitrogen content, Mr_N is the molar mass of nitrogen, Mr_{PAAm} is the molar mass of PAAm, $Mr_{P\text{-}x}$ is the molar mass of PAAm-X, $Mr_{A\text{-}x}$ is the molar mass of the aldehyde and Mr_{water} is the molar mass of water. The polymers are not completely soluble in any water-free solvents, hindering further confirmation of the degree of substitution for example by NMR spectroscopy.

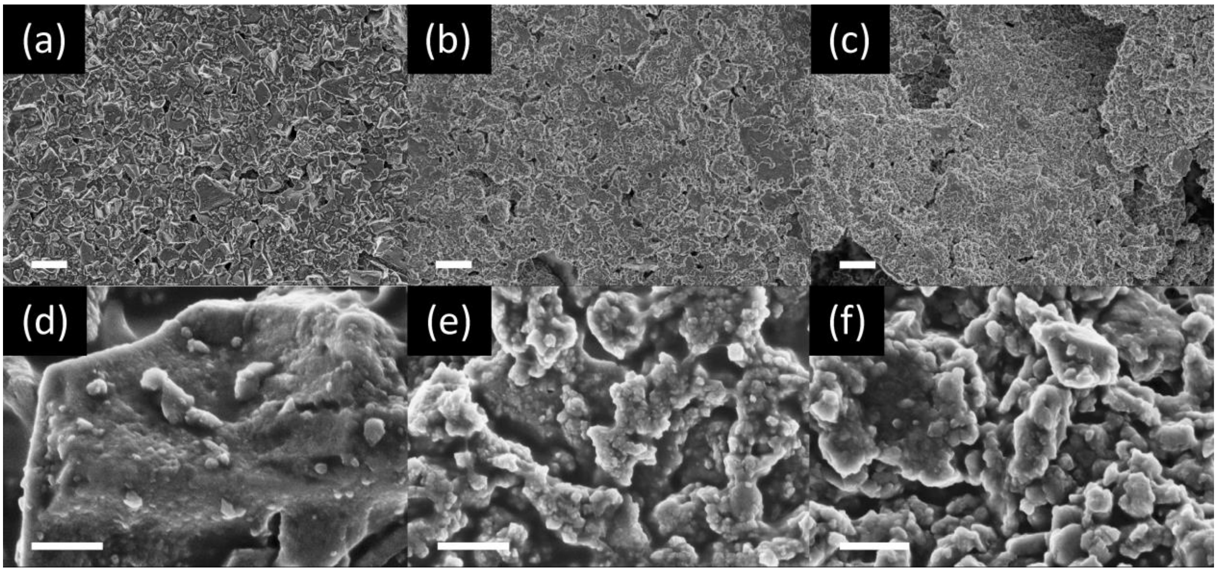

Figure S3. SEM images of C(pristine) (a, and d), C (b, and e), and C/P-o (c, and f). The scale bars represent 10 µm (a, b, and c) and 1 µm (d, e, and f), respectively.

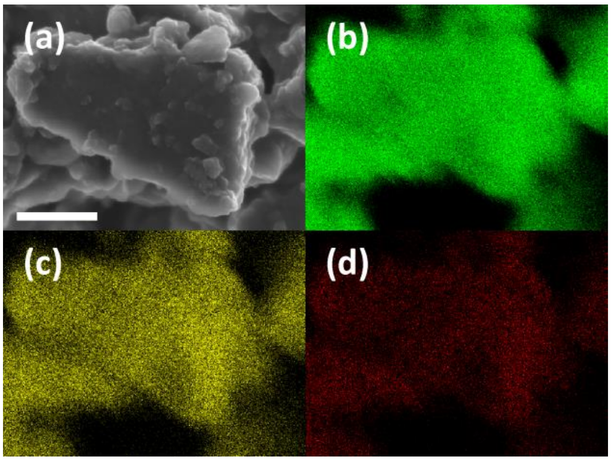

Figure S4. SEM image of C/P-o (a; scale bar 1 µm) and carbon (b), oxygen (c), and nitrogen (d) mapping by EDX.

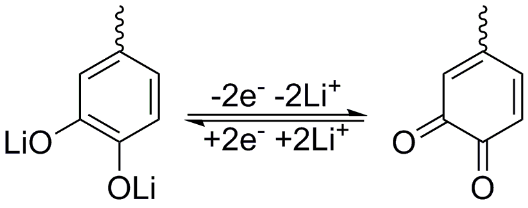

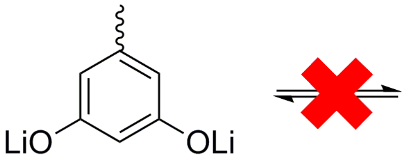

Scheme S2. Redox activity of o-dihydroxybenzoic groups and inactivity of m-dihydroxybenzoic groups.

C/P-o was deposited on carbon paper current collectors and tested as cathode in lithium ion batteries (details can be found in the Experimental Section).

Table S2. Masses of the electrodes used in this study.

Sample Name	Experiment	$m_{Electrode\ 1}$ /mg	$m_{Electrode\ 2}$ /mg	$m_{Electrode\ 3}$ /mg
**C/P-o(20%)**	CDC 50 cycles @ 0.2 A g^{-1}	2.07	2.11	2.07
**C/P-o(20%)**	CV@5 mV s^{-1}	2.07	N/A	N/A
**C/P-o(30%)**	CDC 50 cycles @ 0.2 A g^{-1}	2.02	2.09	2.11
**C/P-o(30%)**	CV@5 mV s^{-1}	2.11	N/A	N/A
**C/P-o**	CDC 50 cycles @ 0.2 A g^{-1}	1.92	1.93	1.82
**C/P-o**	CV@5 mV s^{-1}	2.03	N/A	N/A
**C/P-o(50%)**	CDC 50 cycles @ 0.2 A g^{-1}	1.45	1.45	1.27
**C/P-o(50%)**	CV@5 mV s^{-1}	1.33	N/A	N/A
**C/P-m**	CDC 50 cycles @ 0.2 A g^{-1}	2.28	2.16	2.16
**C/P-m**	CV@5 mV s^{-1}	2.17	N/A	N/A
**C/P-van**	CDC 50 cycles @ 0.2 A g^{-1}	1.46	1.38	1.53
**C/P-van**	CV@5 mV s^{-1}	1.37	N/A	N/A
**C**	CDC 50 cycles @ 0.2 A g^{-1}	1.66	1.83	1.76
**C**	CV@5 mV s^{-1}	1.58	N/A	N/A

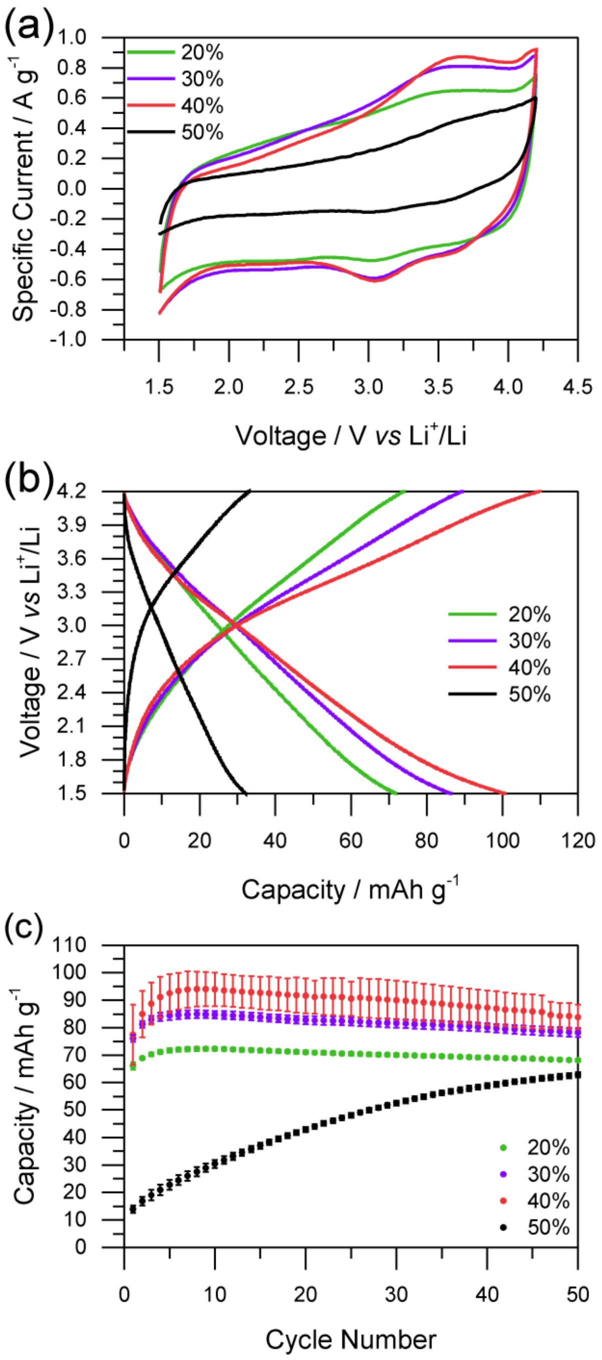

Figure S5. Electrochemical performance of different hybrid materials of P-o polymers with carbon and binder (C/P-o), where the percentage represents the amount of P-o, while the amount of PVDF is constant (10%), and the rest is conductive carbon. The test was performed in a lithium half-cell setup with lithium as counter electrode and 1 M $LiPF_6$ in EC/DEC (1/1) as electrolyte. (a) Cyclic voltammetry at 5 mV s^{-1}, 10th cycle. (b) Charging-discharging test at 0.2 A g^{-1}, 10th cycle each. (c) Discharging capacities as calculated from charging-discharging tests at 0.2 A g^{-1}.

In order to optimize the composition of P-o and conductive carbon multiple hybrid materials were prepared and compared electrochemically using cyclic voltammetry and charging-discharging tests (Figure S5). Capacity in the hybrid materials gradual increases from P-o contents between 20 % and 40 % and significantly decreases when increasing the amount of P-o to 50%. This behavior can be explained by a combination of gradual increase of electrochemically active groups as well as hydrophilic groups (Figure S5a,b) and sharp reduction of conductivity as a result of high content of nonconductive polymer. Additionally, combinations with a maximum of 40 % active polymer material achieve maximum capacity after only a few cycles while the material with more active polymer percentage does not achieve the full capacity even after 50 cycles (Figure S5c). This behavior may be ascribed to reorganization of polymer-bound functionalities that happens upon cycling, allowing for a

better conductivity between carbon and polymer and subsequently higher utilization of redox-active groups and wetting of the carbon material with the electrolyte. Due to the peak performance of 40% composite, all the further experiments with all the polymers have been performed using 40% of the polymer, 50% of conductive carbon, and 10% of PVDF.

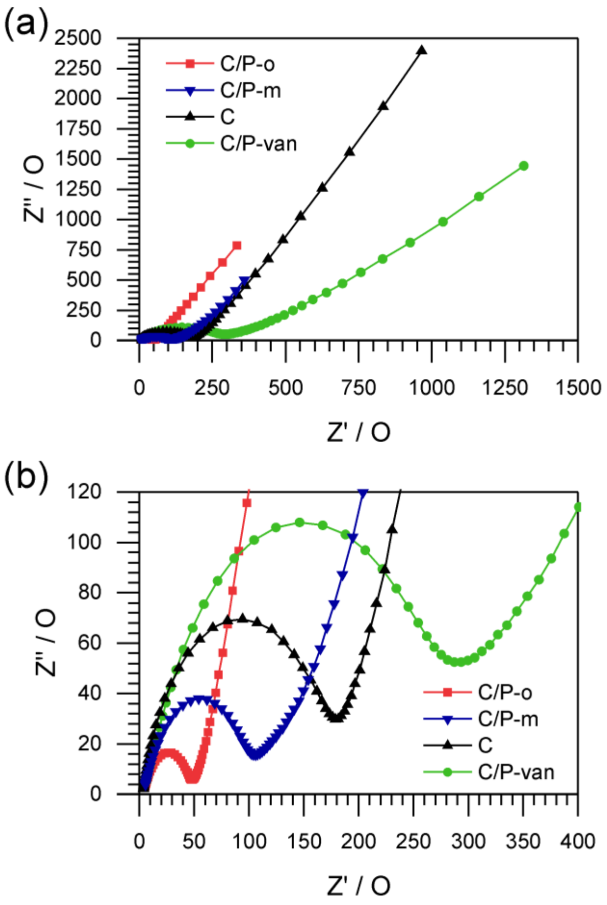

Figure S6. EIS of C/P-o, C/p-m, C, and C/P-van. (a) Whole region. (b) Enlarged.

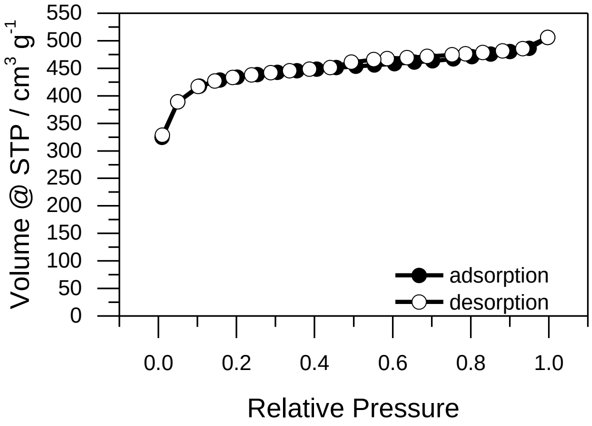

Figure S7. Nitrogen physisorption measurement of C(pristine).

Quantification of different contributions to capacity of P-o

Contributions to charge storage (i) of the conductive carbon’s electrical double layer, (ii) of the influence of enhanced hydrophilicity of conductive carbon, increasing its accessibility by the

electrolyte and thus facilitating capacitive charge storage, and (iii) of the redox activity of *o*-quinone functionalities was calculated by comparing the capacities of C, C/P-m and C/P-o. Capacity of C, divided by 9/5 (as there is 90% of conductive carbon in C compared to 50% in C/P-m and C/P-o), was divided by the total capacity of C/P-m in order to obtain the percentage contribution of the capacitive charge storage of ball-milled carbon. Furthermore, the difference between capacities of C/P-o and C/P-m was divided by the capacity of C/P-o to obtain the percentage contribution of redox activity. The rest of capacity is due to enhanced formation of an electrical double layer of carbon that becomes accessible to the electrolyte due to enhanced hydrophilicity.

Calculation and discussion of the theoretical capacity

According to elemental analysis, P-o is characterized by a substitution rate of 84.4%. Therefore, it is possible to calculate the average molar mass of the repeating unit to be 158.46 g mol^{-1}; however only 84.4% units are redox active. The theoretical capacity of such a polymer equals (where *F* is Faraday constant):

$$C_t = \frac{2 * F}{158.46\ g\ mol^{-1} * 0.844} = 400.8\ mAh\ g^{-1}$$

However, considering that all capacities in this study are referred to the total mass of an electrode, including carbon and binder, and only 40% of the electrode material accounts for redox active polymer, the theoretical capacity deriving from redox reactions of such an electrode is 160.3 mAh g^{-1}. This number is still significantly higher than as obtained in this work. Lower capacity than C_t can be ascribed to a nonoptimal interface between the polymer and the conductive matrix that inhibits the flow of electrons, blocking part of the redox active centers to exchange electrons. Furthermore, solubility to a low extent in the electrolyte and traces of water, leading to detaching redox active groups from the polymer, may result in comparably low capacity and limited stability.

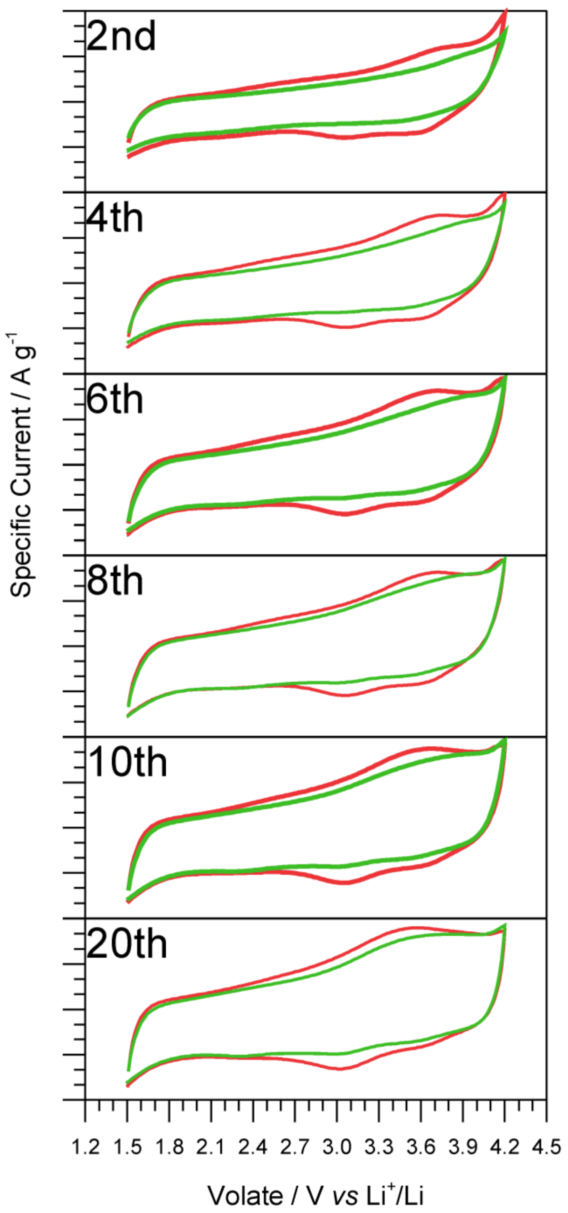

Figure S8. Cyclic voltammetry of C/P-o and C/P-van at 5 mV s^{-1}, selected cycles. The test was performed in a lithium half-cell setup with lithium as counter electrode and 1 M $LiPF_6$ in EC/DEC (1/1) as electrolyte.

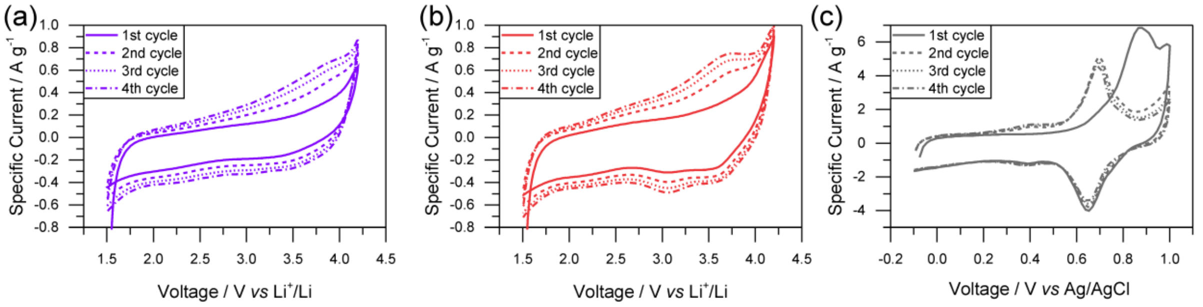

Figure S9. First four cycles of cyclic voltammograms of C/P-van (a), C/P-o (b), and ChiVan-CB (c). Cyclic voltammograms were measured at 5 mV s^{-1} in a lithium half-cell setup with lithium as counter electrode and 1 M $LiPF_6$ in EC/DEC (1/1) as electrolyte (a ,b) or in a three electrode system with platinum wire as counter electrode, Ag/AgCl in saturated KCl as a reference electrode, and 1 M $HClO_4$ as electrolyte (c). More details about ChiVan-CB can be found in ref. [2].

ChiVan-CB – A hybrid material consisting of approximately 77% highly porous carbon and 23% reduced Schiff base copolymer of vanillin and chitosan. It bares guaiacol groups, just like P-van. As the Schiff base is reduced in ChiVan-CB, the material is stable in aqueous environment – in contrast to P-x polymers. Because of this stability, if could be investigated in an aqueous electrolyte and was used instead of P-van here.

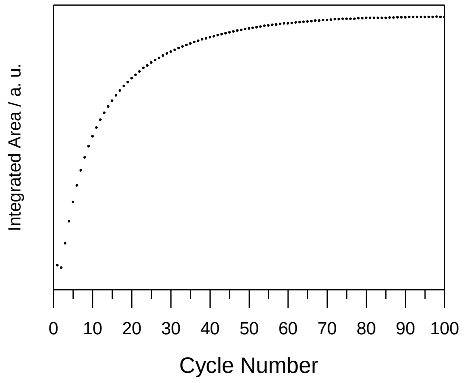

Figure S10. Integrated area of cyclic voltammograms of P-o at 25 mVs^{-1}.

The current response (I) at a certain potential can be separated into two contributions: surface controlled (proportion to scan rate *v*) and diffusion controlled (proportional to square root of scan rate SQRT(*v*)). Upon linearization of this reaction, one obtains:

$$\frac{I}{\sqrt{v}} = k_1\sqrt{v} + k_2$$

Therefore, by measuring the current response at different scan rates one can calculate diffusion and surface controlled current response and therefore the ratio of Faradaic charge storage (due to the redox-active species on the surface, surface controlled) and double-layer capacitance (due to the electric double layer formed, diffusion controlled).

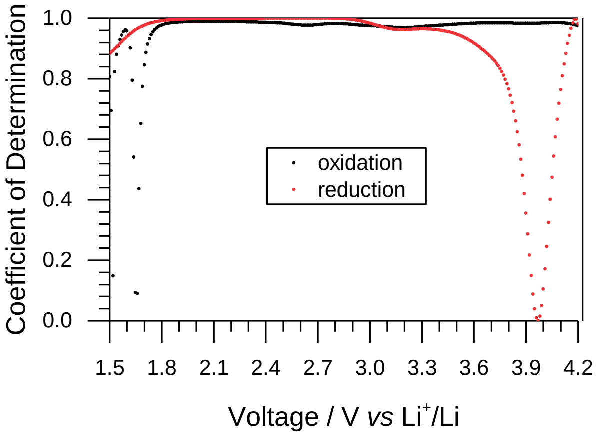

Figure S11. Coefficient of Determination (*aka* R^2 value) for calculation of Farradaic and non-Farradaic contributions to cyclic voltammetry as calculated from data presented in Figure 4c.

References

1 E. Oikawa and K. Yahata, *Polym. Bull.*, 1987, **17**, 315–322.

2 I. K. Ilic, M. Meurer, S. Chaleawlert-umpon, M. Antonietti and C. Liedel, *RSC Adv.*, 2019, **9**, 4591–4598.
